# Experiment and modeling of concentration-dependent diffusion in a solution with dual transport-phase flow for paper-based sensing†

**DOI:** 10.1039/d5ra02347e

**Published:** 2025-06-04

**Authors:** Md. Saykat Hassan Sajib, Md. Sakif Rafid, M. Ryyan Khan

**Affiliations:** a Department of Electrical and Electronic Engineering, East West University Dhaka Bangladesh ryyan@ewubd.edu

## Abstract

Paper-based sensors are promising as low-cost, passive devices for point-of-care clinical diagnostics, food quality assessment, and environmental monitoring. The diffusion process in paper-based sensors, and component detection through multistage transport or multiphase solution flow (*e.g.*, in chromatography) have been studied in literature. However, observation or analysis of concentration dependent analyte flow in such systems has not been considered. In this work, we performed a set of experiments on concentration dependent liquid-flow in paper strips, developed a video processing technique for automatic fluid-flow-distance detection, and explained the observation through a mathematical model. We use KMnO_4_ solutions – such solutes which have weak bonds with water (solvent) can have decoupled transport-phases: (i) water flow, followed by (ii) analyte diffusion. We have presented a video processing algorithm to automatically extract the time-series analyte and water flow distance measurements by analyzing the pixel values frame-by-frame of the recorded experiment videos. This ensures consistency in distance measurements among the experiments. We finally explain the physical process of the flow using a corresponding mathematical model to describe the concentration effects in paper-like materials. The model includes how analyte undergoes drift force (due to water flow velocity) along with diffusion. We found that the numerical solution of the model agrees with the trends seen in the experimental results. This can help us better understand the liquid-wicking behavior in different concentrations through a mathematical model and provide guidance in the design and optimization of paper-based sensors.

## Abstract 2

(A) Video processing to automatically detect fluid-flow-distance. (B) Concentration dependent two-transport-phase flow distance explained using experiments and a mathematical model. (C) Studied for different source conditions and device orientations.

## Graphical abstract

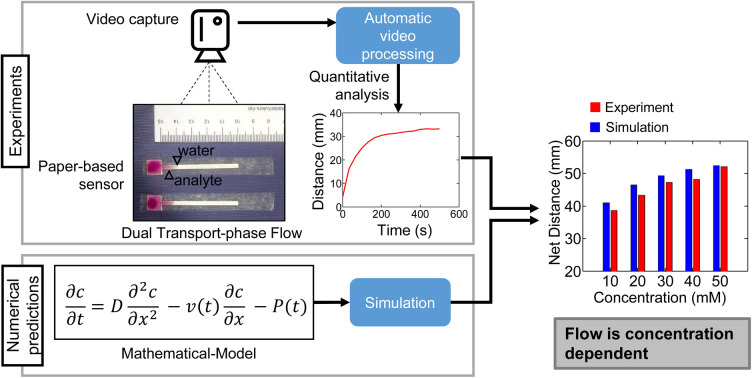

## 1. Introduction

Over the last few decades, paper-based sensor technologies have shown great promise due to its lower cost and simpler implementation. There have been significant progress in relevant fabrication, fluid control techniques for sample processing and analysis. Paper-based devices are widely used for medical diagnosis such as simple blood sugar level and protein testing, urine testing to measure pregnancy hormone or glucose contents,^1^ and DNA detection.^2^ Paper-based devices can also be used to monitor food quality and preservation,^3^ and for environmental monitoring of chemicals and pollution in air^4^ and water.^5^ The fundamental physical process of these applications is mass transfer within the porous structure of paper-like materials, where liquid-wicking behavior by capillary action plays a significant role. It is crucial to clearly understand the fluid flow behavior and fluid control mechanisms to establish a framework for designing paper-based diagnostics or sensing devices.^6^

### 1.1. Fluid control (device design)

Various fluid control methods have been proposed to improve the sensitivity of paper-based devices, such as changing geometrical structures, making hydrophobic patterns, adding moderator reagents, and so on. In geometric control strategies, different wicking behavior is achieved by changing the dimension of the paper strips. The simplest geometric control is a distance-based delay. For instance, Fu *et al.* investigated liquid flow in multiple connected straight paper strips with varying widths to control wicking times to achieve multi-step reactions in one paper strip.^7^ Apilux *et al.* created a serpentine channel by printing baffles on the paper to increase the wicking length that delayed flow.^8^ These studies showed how to improve the sensitivity by controlling fluid speed by chaining the geometrical structure of paper strips. Besides changing paper structure, the porous structure of papers can also be modified with hydrophobic material (*e.g.*, paraffin wax) or dissolvable barriers (*e.g.*, sugar and trehalose) to slow down fluid speed directly. Toley *et al.*^9^ and Shin *et al.*^10^ pass the fluid through a dissolvable barrier (known as a shunt) to obtain a flow delay, which is added onto paper strips to reduce the available wicking area. Lutz *et al.* deposited different concentrations of sugar into paper strips to create flow delays for an automated multi-step assay device.^11^ Noh *et al.* demonstrated a 3-D vertical flow assay, where various amounts of paraffin wax were deposited into paper strips to create delay. They found that paraffin wax could generate a delay of several minutes.^12^ Additionally, Camplisson *et al.*; Channon *et al.* accelerated flow by creating two-ply channels.^13,14^ They created a hollow region in the center of the channel by sandwiching two paper strips between hollow layers of double-sided tape, which has much lower fluid resistance compared to the paper around it. Renault *et al.*; Shin *et al.* created ‘macro-capillaries’ for the liquid to flow through by removing regions of the paper to create faster wicking channels.^15,16^ Other groups Giokas *et al.* and Liu *et al.* etched grooves into paper using a craft plotter, and CO_2_ laser cutter respectively to speed up flow.^17,18^ The different fluid control strategies on paper-devices discussed above are mostly developed experimentally through trial and error. The simplistic theoretical imbibition models are however unsuitable for complex device design and application conditions.

### 1.2. Concentration detection

Several works have experimentally shown concentration dependent variation in solution flow in paper based sensors. For example, Mirzaei *et al.* proposed a distance-based method for the detection of blood lactate concentration using a paper strip coated with gold nanoparticles (AuNPs).^19^ As the lactate flows through the paper strip, it interacts with the nanoparticles and changes color from red to purple. The lactate concentration was detected by distance analysis. The researchers found a linear relationship between the color length and lactate concentration in the range of 1.0 to 30.0 mM. A similar method was used by Phoonsawat *et al.* to determine chloride ions (Cl^−^) in water by functionalization of silver nanoparticles (AgNPs) on the paper channel.^20^ Soda *et al.* introduced an ion-selective capillary sensor, which can determine the potassium ion (K^+^) concentration.^21^ They demonstrated that ion-selective membrane films cast on the inner wall of the capillary channel can respond sensitively to a narrow range of K^+^ concentrations (1.0–6.0 mM), and can be used in a sensing mode. Moreover, Patari *et al.* detected the presence of added water in milk by measuring changes in capillary height resulting from varying water concentrations. They added 25%, 50%, and 75% water to pure milk and measured changes in capillary height. As the water content increased, the capillary height increased as well.^22^ Xu *et al.* modified paper cellulose by depositing water-insoluble oxidants onto the paper channel to create a distance-based analytical device for detecting reducing substances.^23^ Using different concentrations of ascorbic acid and captopril solutions, they established a calibration curve and observed a linear relationship between the concentration and the length of the discolored band. These works, however, do not provide theoretical or mathematical explanation of the concentration dependent fluid flow.

### 1.3. Fluid flow physics

Besides experimental studies, the mathematical model also plays an important role in predicting liquid-wicking behavior for better fluid control in the paper. Lucas–Washburn (L–W) model has been widely used in the study of one-dimensional liquid-wicking behavior in porous materials.^24^ They defined a relationship between liquid wicking distances (*h*) with time (*t*) as 
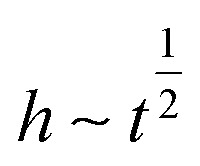
, which showed good agreement with experimental results. However, this model ignored some key factors such as characteristics of materials, evaporation, gravitational force, channel boundary effects, and the environmental temperature and humidity which are significant in real applications. Consequently, this model cannot predict the experimental results of the liquid-wicking phenomenon in porous materials over long time intervals. Therefore, various modified models were developed considering different factors to give better descriptions. Ponomarenko *et al.* developed a general relationship between liquid front and time considering the gravitational force because it is an important factor for long-term liquid-wicking behavior.^25^ The relationship showed that liquid imbibition height has a power-law relationship with time as 
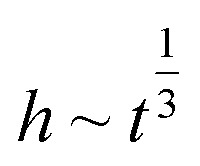
. Li *et al.* proposed the criteria for using the L–W model in the case of spontaneous imbibition of a liquid in porous media.^26^ They also described the criteria for including the effect of gravity on wicking using the CGR number (capillary pressure-to-gravity force ratio). Besides, Fries *et al.* modified the L–W model to account for the evaporation effect in the wicking of different liquids in porous media.^27^ They showed that capillary rise is inversely proportional to the evaporation rate. Liu *et al.* also developed an evaporation model in the case of paper matrix to predict liquid wicking height and mass by varying the width of paper strips.^28^ Hong and Kim proposed an empirical model to investigate the wicking behavior in hydrophobic channels with various boundaries.^29^ They illustrated a relationship between hydrophobic channels and the dynamic wicking behavior in channels. This relationship showed a novel strategy to decrease the liquid velocity by adjusting the contact angle with the wax boundaries. These models are focused towards studying wicking behavior in porous materials with 2D or 3D complex geometric structures and the effects of micro structural parameters of fibrous materials such as porosity, fiber diameter, *etc.*

Considering properties of the media such as porosity and permeability, Darcy first described fluid flow in porous materials.^30^ He found that the flow rate in porous media is proportional to the pressure drop through the porous medium. This relationship is known as Darcy's law. The electrical circuit analogy (ECA) model has also attracted significant interest because it is a simple approach for modeling the fluidic network by electric circuits. Fu *et al.* calculated the transport time for flow through a paper strip with different widths using Darcy's law by analogy to Ohm's law.^7^ Furthermore, the ECA approach has been used to model different paper networks that have utilized various materials such as a sponge shunt integrated on lateral flow assay (LFAs) with varying contact angles and coated an absorbent shunt on a strip.^9,31^ However, these models did not consider the effect of concentration on the wicking process in the paper matrix. Piquemal *et al.* modeled species movement in porous media using a dispersion–convection equation for the mobile fluid and a diffusion equation for the stagnant fluid (two-stage transport). Their study focused on a cylindrical tube with stagnant regions along its walls and also explored the behavior in a layered permeable medium.^32^

Such two-transport-phase flow in porous medium is regularly utilized in chromatography. The concept of chromatography has been appended with multiple techniques for applications in different applications. For example, Masanobu Motooka *et al.* improved gas sensing using chromatography paper for breath analysis, with potential diagnostic applications such as diabetes screening.^33^ Patrick Ryan *et al.* developed a graphite electrode-based electrochemical probe integrated to chromatography paper using ethylene glycol-choline chloride electrolyte for sensitive TNT detection in soil.^34^ Xiao-Lei Huo *et al.* integrated electrochemiluminescence (ECL) with paper chromatography for simultaneous separation and detection of (bisphenol A, BPA, and dibutyl phthalate, DBP) for multi-target environmental analysis.^35^ The underlying mechanism of multi-stage transport and multi-phase fluid dynamics and its applications have been discussed in the literature.^36^ Fei He *et al.* numerically investigated the transient two-phase fluid flow with liquid phase transitions through micro- and nanoscale porous medium.^37^

There are prior works on paper-based or chromatography-like systems with well established models^38^ explaining the physics of the underlying diffusion process. However, there is no analysis of concentration dependency and its potential detection in the multi-stage-transport or multi-phase flow systems on nonfunctionalized paper channels.

### 1.4. This work

In this paper, (i) we develop an automatic fluid-flow-distance detection method by video processing, (ii) through experiments and a mathematical model, we explain how the wicking distance of a two-transport-phase flow shows concentration dependency, and (iii) study how the behavior may change for different source conditions and device orientations. To elaborate: we present a detailed time-series experiment of solution flow through paper strips for various concentrations. To measure the experimental observations of fluid flow, we implement a video processing algorithm for robust and consistent extraction of time-series analyte flow distance through the paper channel. We choose KMnO_4_ solutions for the experiment—this shows a 2-stage transport where water flows faster than the purple MnO_4_^−^ ions. The detailed experimental measurements (time and distance) for concentration dependent solution flow yields in estimation of solvent (water) velocity and analyte flow distance. We finally present a convection–diffusion equation-based, physically relevant mathematical model representing this system. Our mathematical model and numerical analysis agree with the trends observed in the experiments. Such mathematical models to represent the concentration effect are vital for the design and performance analysis of paper-based sensor or diagnostic devices.

## 2. Concept/problem statement

Most paper-based sensors are explained using some form of L–W model. According to L–W model,^24^ we do not expect variations in fluid or solution flow distance through paper-channels for different concentrations. This is consistent with several experiments in literature. For some solutions, however, a concentration dependent solution flow distance have been observed.^22,23^ It is unclear how or why such dependencies occur. In case of solute with weak bonds with the solvent, there can be a two-stage transport. For such cases, the solvent (*e.g.*, water) flows through the paper channel first, and then the solute (*e.g.*, KMnO_4_) shows a secondary diffusion (two transport-phases). Although two transport-stage flow models have been discussed in the literature,^32,36^ concentration dependency has not been explored. We hypothesize that a velocity assisted diffusion can have concentration dependencies. We will test this hypothesis, and explain it using a general mathematical model describing this process.

## 3. Experimental setup

### 3.1. Paper sensor structure

In most of the research studies, Whatman filter paper is used because it is cheap, thin, easily available, lightweight, easy to store and transport, disposable, and biodegradable.^39^ We used Whatman no. 1 filter papers because of its homogeneous and hydrophilic nature. A common problem in paper-based device is the reduction in wicking speed due to evaporation. Therefore, we sandwiched (laminate) the paper between polymer films to prevent liquid leakage and evaporation during flow. A thin strip of laminated paper works as the fluid flow channel for the paper based sensor.

### 3.2. Device preparation

**Fig. 1:**
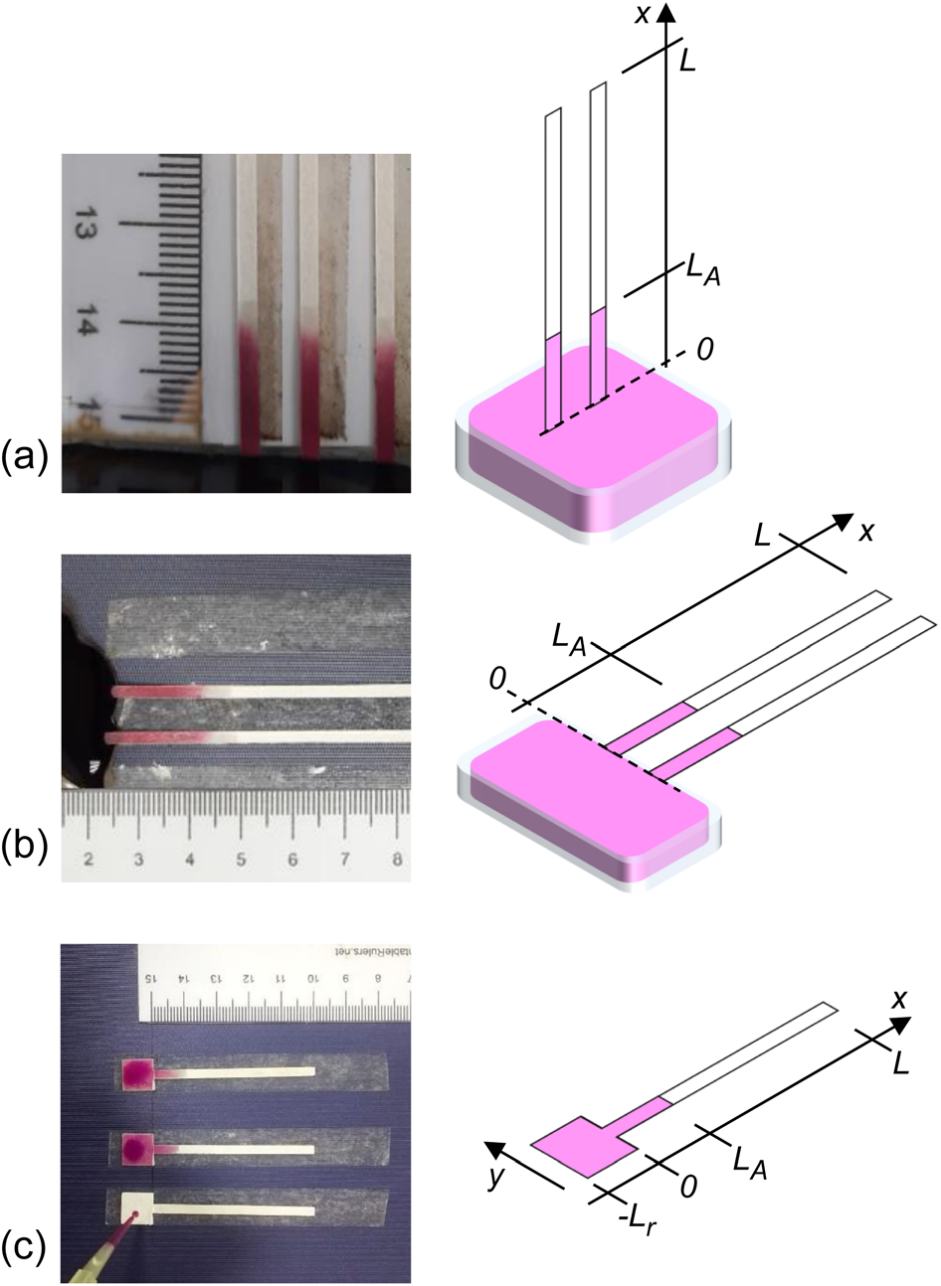
Experimental setup and the corresponding schematic diagrams for (a) infinite-source with vertical strip (IVS) (b) infinite-source with horizontal strip (IHS) (c) finite-source with horizontal strip (FHS) configurations.

The polymer film was attached to the filter paper on both sides using a laminator (JL330T, PFEC) at 130 °C with a feeding speed of 1 cm s^−1^. The laminated filter paper was cut to make the channels of the devices using a laser cutting machine (R500 Laser cutter, Rayjet) according to the design of the paper-sensor (device). The paper strip has two parts (see Fig. 1(c)): a reservoir (10 mm × 10 mm) where the liquid was deposited and a straight channel to flow the liquid with a width of 3 mm and a length of channel 80 mm. A double-sided tape was used to attach the prepared device to a flat surface. Additionally, a scale bar was placed alongside the paper strips to calibrate and measure the length of the liquid front during video and image processing. We will use different concentrations of aqueous solutions of KMnO_4_ as the working fluids in various tests. This purple solution will facilitate the development of distance-based detection on paper-based microfluidics device aiming to quantify analyte concentration based on color length.

### 3.3. Experimental process

The experiments were conducted in different ways depending on the positioning of the paper and solution source (*i.e.*, solution reservoir) to examine the effects of concentration on liquid-wicking behavior in paper-like materials: (i) infinite source: vertical strip; (ii) infinite source: horizontal strip; (iii) finite source: horizontal strip.

#### 3.3.1 Infinite-source with vertical strip (IVS)

For this case, the paper strips were vertically positioned with a stand and dipped into a Petri dish filled with the solution – see Fig. 1(a). The volume of the solution source is much higher than the amount that can be wicked into the paper strip. Hence the name: infinite source. A mark is placed on each of the paper strips to indicate the dipping level in the liquid. The liquid wicks up through the paper strips due to the capillary force.

#### 3.3.2 Infinite-source with horizontal strip (IHS)

The paper device was adhered to a flat surface using double-sided tapes, placed horizontally, and one of its edges was placed on the liquid sample for wicking – see Fig. 1(b). The paper strips were marked to indicate the dipping position. When the paper strip mark touched the sample surface, we started our measurements (through video recordings).

#### 3.3.3 Finite-source with horizontal strip (FHS)

The experimental procedure involved adding a 30 μL solution with a specified concentration to the center of the reservoir of the horzontally placed paper device – see Fig. 1(c). Due to the capillary action, the solution flowed from the reservoir and then along the paper channel.

Every experiment was run multiple times for each specific concentration to average out variability – see Sec. S1 in the ESI† document for relevant details. Fluid-flow into the paper channel was video-recorded for 20 minutes after introducing the solution at the paper channel. The position of the fluid flow wavefront was obtained by video and image analysis methods. See Table 1 for a summary of the experiments.

Summary of experiments

**Table:** 

Items	Details
Device material	Whatman no. 1
Channel size	3 mm × 80 mm
Reservoir size	10 mm × 10 mm
Imaging system	Camera (smartphone), fixture or mount, post-processing algorithm
Solution	KMnO_4_ in water
Concentrations	10, 20, 30, 40, and 50 mM

**Table:** 

Experiments	Type	Source volume	Channel orientation
	IVS	Infinite	Vertical
	IHS	Infinite	Horizontal
	FHS	Finite	Horizontal

### 3.4. Data extraction

**Fig. 2:**
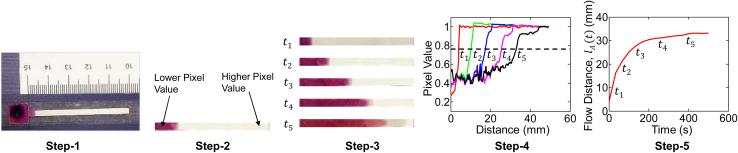
Position and time resolved data extraction steps are shown: (1) video recording, (2) frame alignment and scaling, (3) sample video frames for analysis, (4) extracted spatial profiles of intensity (representative of local concentration), (5) analyte flow distance with time.

We developed an algorithm to extract data from the recorded experimental video. The video was imported into the computer, and data was extracted from each frame to determine the distance flowed by the analyte front. Use of an algorithm eliminates human error and ensures consistency among the information extracted from all the experiments. The calculation steps followed in the algorithm is summarized in Fig. 2.

Step-1: each of the experiments were video-recorded. The camera was fixtured vertically away from the paper-device plane. As a reference for physical length, a scale bar was placed alongside the paper-devices within the video capture frame. The video is then loaded into a computer program for frame-by-frame analysis. The pixel dimension of the video was converted to millimeters by calibrating to the scale in the video frame. This would need to be done in the first frame of each video. The calibrated value can vary for different experiments (*i.e.*, different videos). For our various sets of experiments and repetitions, we had found values ranging between 6 and 13 pixelsper mm. All our video captures were at 30 fps (which can also be extracted from the metadata of a video file).

Step-2: the analysis region of interest is the paper channel where the analyte will flow. The video frame is therefore appropriately rotated and cropped to this region (starting beyond the reservoir). This is only needed to be done in the first frame – the selected region will automatically remain the same, given that the camera fixture was sturdy. The part of the channel where the analyte (*i.e.*, KMnO_4_) has diffused is dark, and represented in the video frame as a low pixel value.

Step-3: the channel image is extracted for each frame of the video (see sample images shown in step-3 in Fig. 2). Each of the frames are converted to gray-scale for further processing. The rightmost part of the channel is never wicked and always has the highest pixel value (see step-2 in the figure). Each of the video frames are normalized to this high pixel value to correct for possible temporal fluctuations in ambient lighting.

Step-4: at each frame, the pixel values are averaged along the width (*i.e.*, along *y*-axis, also see Fig. 1) to get a 1D intensity matrix along the channel length. For example, such plots for 5-different time frames are shown in step-4 of Fig. 2. The pixel values are low (*i.e.*, dark wetted part) along the channel, followed by a clear transition to brighter pixels (*i.e.*, unwetted part). The transition obviously marks the flow front of the analyte. For consistency among all experiments, we set a threshold pixel value of 0.75 (marked by dashed line in the figure) to mark the analyte flow distance at a given time frame. The water flow front was detected by taking a spatial derivative of the pixel value *vs.* distance curve and following the corresponding transition peak – see Sec. S2 in the ESI† document for full details.

Step-5: the flow distance estimated for each time-frame found in the previous step is collated to form the flow-distance *versus* time relationship.

The sample time-series analyte and water flow distance extraction videos provided as additional ESI† demonstrate the algorithm. In Sec. S5 of the ESI† document, we have also illustrated how the 5-step process works on the video frames.

The post-processing of the captured video (*i.e.*, steps 1–4) is such that the data extraction process works consistently regardless of the imaging system or camera (any DSLR or smartphone camera would be fine). The only requirement is that the video capture should have a resolution high enough to detect the flow front at each time step.

## 4. Results: data analysis

Aqueous solutions of KMnO_4_ (analyte) were prepared with concentrations of 10, 20, 30, 40, and 50 mM. The experiment steps were repeated for each concentration, for each of the experimental setups (discussed in the previous section). Multiple paper strips were used in each experiment setup and concentration combination. For a given setup, the capillary flow distance *versus* time data is averaged over the multiple runs of each concentration.

**Fig. 3:**
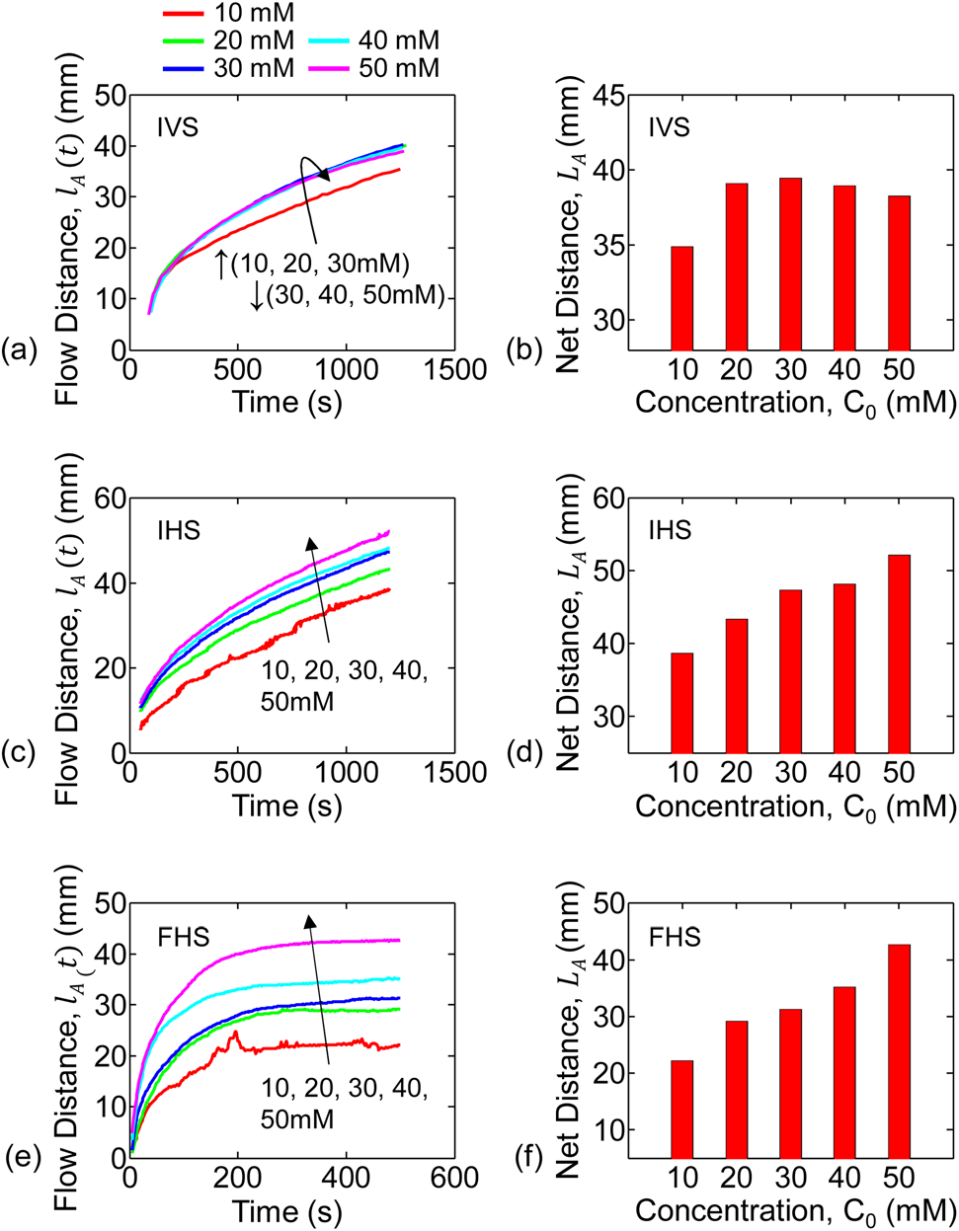
Analyte flow distances through the paper-channel extracted from the experiments are shown for various concentrations. Continuous analyte flow distance (*l*_A_(*t*)) *versus* time for IVS, IHS, and FHS are shown in (a, c and e). Also, the end-of-experiment net flow distance *versus* concentration for IVS, IHS, and FHS are summarized in (b, d and f).

For the infinite source with vertical strip (IVS) setup, the analyte (*i.e.*, KMnO_4_) flow distance *versus* time for each concentration is shown in Fig. 3(a). The flow distance after 1200 s for various concentrations are shown in Fig. 3(b). It is observed that the analyte distance increased with concentration from 10 mM to 30 mM and then decreased for 40 mM to 50 mM. This can be due to the combined effect of gravitational force and inconsistent flow velocity of the liquid solvent (*i.e.*, water). We only observed the inconsistent water flow velocity in the vertical strip setups. This is further discussed in Section 5.2 and in Sec. S3 of the ESI† document.

The temporal change in flow distance and the final distance (at 1200 s) for various concentrations for the infinite source with horizontal strip (IHS) is shown in Fig. 3(c and d). The corresponding results (at 500 s) for finite source with horizontal strip (FHS) is in Fig. 3(e and f). Clearly, as seen in Fig. 3(d and f), the flow distance increases with concentration. However, there is a distinctive characteristic difference in analyte imbibition with time for IHS and FHS setups as observed from Fig. 3(c and e). For IHS, the analyte flow distance continuously increases with time. On the other hand, the analyte imbibition distance saturates after some time for FHS. We hypothesize that in FHS setup, the imbibition distance is limited because the solution volume and hence the analyte is finite. In the following section, we will explain the trends of the experimental data of the analyte flow using our mathematical and numerical model.

## 5. Model and interpretation

### 5.1. Numerical model for two transport-phase flow

Mass transport by capillary action through porous media exhibits diffusive behavior. When a KMnO_4_ crystal is placed into the water, it dissolves into K^+^ and MnO_4_^−^ ions. The MnO_4_^−^ ion's purple color spreads throughout the water. When the KMnO_4_ solution contacts with the paper strip, we observe that water flows through the paper channel first, followed by a secondary flow of purple analyte (MnO_4_^−^ ions). Such a solution flow with two transport-phases can happen for any weakly soluble chemical or biochemical compounds.^23^ The fluid velocity and molecular diffusion are the two main factors that govern the mass transport of the moving fluid through the porous media in this wicking process. Fick's second law describes how the spatial distribution of concentration changes over time due to diffusion. For the capillary-driven flow, this law can be reformulated as follows:1
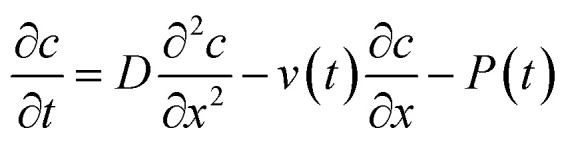
This is known as the convection–diffusion equation. Where *c* is the concentration of the solute, *D* is the diffusion coefficient (mass diffusivity for particle motion) of the solute (or analyte) in the carrier fluid, *v*(*t*) is the velocity of the analyte induced in the carrier fluid, and *P*(*t*) is related to the volumetric source of *c* – this (*i.e.*, *P*(*t*)) represents the generation or reduction rate of *c* within the system.^40^ The concentration of diffusion species (*i.e.*, analyte) is a function of both time and position *c* = *c*(*x*, *t*). For our case, we have *P* ≈ 0. We also assume no net flow along *y* – direction (*i.e.*, vertical to the channel), thereby reducing our practical system to a 1D problem described by eqn (1).

The second term on the right hand side of eqn (1) represents the drift force on the analyte due to the water flow velocity. The overall process can be interpreted as follows: the carrier fluid flows along the channel (transport phase-1) and its velocity enhances the net diffused analyte flow distance (transport phase-2). The dual transport-phase flow is consistent with our experimental observation. The flow and velocity of the carrier fluid (*i.e.*, water in our experiment) along the paper strip can be estimated from mathematical and numerical modeling.^22^ This will however vary with the experimental setups. For our analysis, the water flow velocity was determined by image processing and curve fitting methods described as follows. For a given setup (IVS, IHS, or FHS), it is expected that the water will travel the same distance for all concentrations. We estimated the water flow distance (*l*_w_) *versus* time following a technique similar to that discussed in Sec. 3.4. We found the trends to be of the following form:

2 *l*_w_(*t*) = *L*_w∞_(1 − exp(−*t*/*τ*)).

**Fig. 4:**
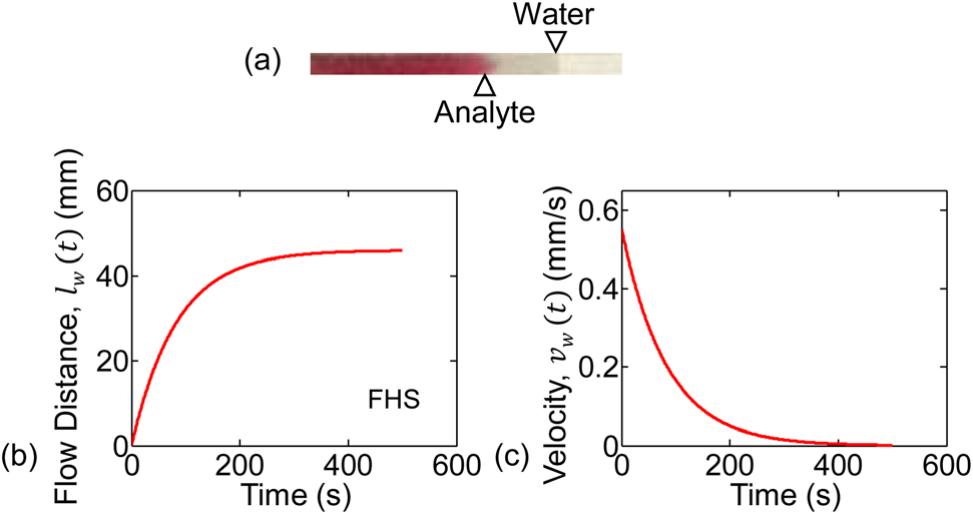
A sample analysis of water flow velocity for FHS is shown. (a) Presents the analyte and water positions on the paper channel at a given time-frame. (b) The curve-fitted water flow distance with time (*l*_w_(*t*)), and (c) the corresponding water flow velocity (*v*_w_(*t*)) is shown.

Here, *L*_w∞_ and *τ* are found through the fitting process discussed above – these will have different values for the different experimental setups. Now, the water velocity is:3
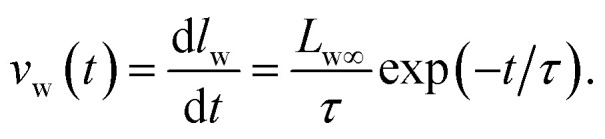
An example plot of water flow distance (*l*_w_) and flow velocity (*v*_w_) *versus* time for FHS is shown in Fig. 4. The induced analyte particle velocity will be lower than *v*_w_ due to drag, *i.e.*, *v*(*t*) = *αv*_w_(*t*). Moreover, in the case of vertical setup (IVS), gravity will work in the opposite direction of the particle flow. We can thus find the analyte particle velocity as: *v*(*t*) = *αv*_w_(*t*) − *βC*_0_. Here, to the zeroth order, we have assumed the effect of the gravitational force to be proportional to the analyte source concentration *C*_0_. Eqn (1) can thus be written as follows:4
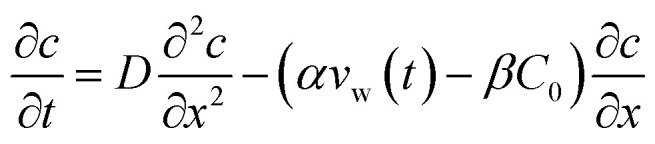
Overall, the remaining free (fitting) parameters in eqn (4) are *D*, *α*, and *β*. Here, *β* can be non-zero only when gravity works along or against the flow direction (*i.e.*, vertical channel). For IHS and FHS, we will have *β* = 0. The diffusion coefficient *D* will be the same regardless of the experiments, while *α* will have a different value depending on the paper position (vertical *vs.* horizontal). The values of *D* and setup-dependent parameters (*L*_w∞_, *τ*, *α*, *β*) are listed in Table 2. The final water flow distance (*L*_w∞_) is low for IVS due to the gravitational forces on the water flowing through the vertical channel. For FHS, *L*_w∞_ is also low as the net volume of water in the source (reservoir) is finite.

List of parameter values used in the numerical simulations. The case with gravitational effects is only applicable for IVS

**Table:** 

Parameter	Value
*D* (mm^2^ s^−1^)	0.07

**Table:** 

Parameter	IVS	IHS	FHS
*L*_w∞_ (mm)	49.95	64.15	45.94
*τ* (s)	383.53	428.89	83.18
No gravity:^a^^,^^b^ (*α*, *β*)	(0.48, 0)	(0.48, 0)	(0.48, 0)

a There is no gravitational effect on the horizontal setups (IHS, FHS). IVS also modeled in this case for comparison.

b With gravity, for IVS: *α* = 0.53, and *β* = 2.1 × 10^−4^ mm s^−1^ mM^−1^.

We assume our system to be represented by a 1D channel (no analyte flow vertical to the channel) of length *L*. The analyte will flow along *x* – direction (channel) with the source at *x* = 0 and the channel terminating at *x* = *L*. Eqn (4) is solved to find concentration distribution along the channel at each time step (*i.e.*, *c*(*x*, *t*)). This same equation will appropriately give results for IVS, IHS and FHS based on the boundary conditions. There are two conditions based on the analyte source: (i) infinite source, and (ii) finite source (mass conservation). We will discuss these in the following.

#### 5.1.1 Infinite source model

In this case, the volume of the sample solution is not limited, and will not be depleted as it flows into the channel – this is true for both IVS and IHS. With the solution and analyte source at *x* = 0, the initial and boundary conditions are as follows:
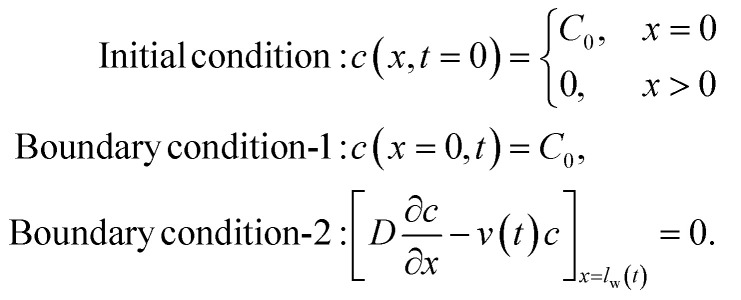
Here, *C*_0_ is the concentration of the solution being tested. These conditions are the same for the IVS and IHS methods; only the carrier fluid (water) velocities are different. Note that, the boundary condition-2 at *x* = *l*_w_(*t*) indicates that the solute cannot flow out of the water at any time. As a negligible amount of the solute reaches *x* = *l*_w_(*t*) (solute is much slower than the water flow front), the boundary condition-2 can be approximated to *c*(*x* = *L*, *t*) = 0 (with only 0.3% difference in the answers).

#### 5.1.2 Finite source model

In the FHS method, we dropped a fixed volume of sample solution onto the reservoir region of the paper device. As the analyte flows from the reservoir and through the channel, the source is depleted (*i.e.*, finite source) and the mass is conserved over time within the closed system.

For the finite source model, we assume a 1D system (with −*L*_r_ ≤ *x* ≤ *L*) where the reservoir is −*L*_r_ ≤ *x* < 0, and the channel is 0 ≤ *x* ≤ *L*. While the water velocity is *v*_w_(*t*) in the channel, we assume it to be zero within the reservoir. The conditions to solve eqn (4) in this case are:
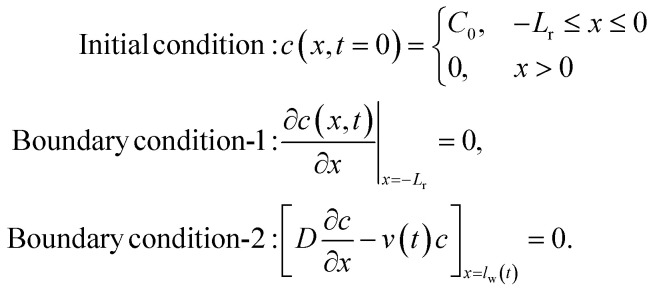
While solving eqn (4) numerically using time-stepping, along with the boundary conditions for FHS, additional calculation may be required for mass conservation, *i.e.*, 
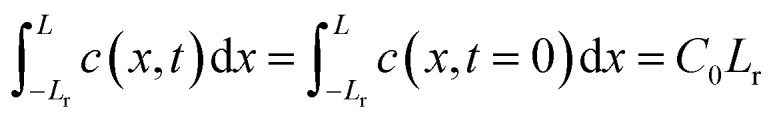
.

In summary, we extracted the water flow dynamics from the experiments and then used the convection–diffusion equation to model the overall analyte flow. We do not explicitly model the wicking of water. The flow of water or an aqueous solution in paper predominantly relies on capillary action, and hence could be modeled using Darcy's law. A more detailed approach would be a 2D transient flow model through a porous medium.^41–43^ However, this is beyond the scope and target of this work and is left for future studies.

### 5.2. Results and interpretation

**Fig. 5:**
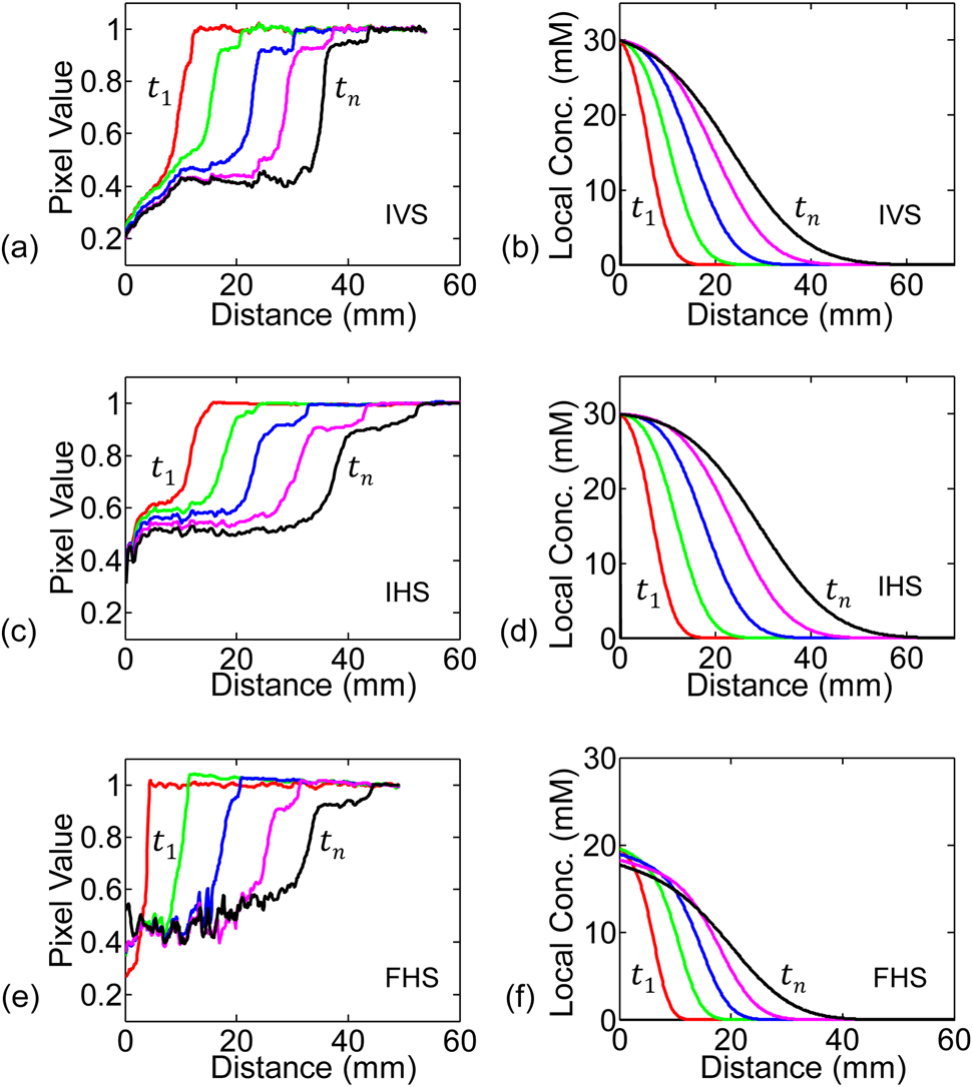
The measured pixel value at a given position is proportional to the observed intensity. Higher pixel value (*i.e.*, brighter spot) indicates lower concentration. (a, c and e) shows spatial profile of pixel value (hence concentration transition) through the paper-channel for IVS, IHS, and FHS experiments. Each curve in (a), for example, represents the measured pixel value spatial profile at each time-step for IVS. (b, d and f) shows the numerically simulated time-stepped spatial profile of concentration through the paper-channel.

We numerically solve eqn (4) following the conditions discussed in the previous sub-section. The spatial and time steps have been set small enough to ensure stability in the solutions. The numerically found spatial distribution of concentration at different time steps (*c*(*x*, *t*)) for 30 mM solution are shown in Fig. 5(b, d and f), alongside the experimental measurements in Fig. 5(a, c and e).

As seen in Fig. 5(b, d and f), for any of the three systems: IVS, IHS, or FHS, we observe that at time *t*_1_, concentration is high near the source reservoir (*i.e.*, near *x* = 0) and then falls off as we move along the channel (*i.e.*, at higher *x*). Higher concentration of KMnO_4_ will be darker (corresponding low pixel value in experiments) and *vice versa*. The transition from dark to bright region marks the flow distance of KMnO_4_. On the other hand, in the simulations, the transition in the local concentration marks the flow distance of KMnO_4_. There is no one to one mapping of the KMnO_4_ local concentration to the darkness (or intensity) measured in the experiments. Regardless, the numerically found concentration profile is conceptually consistent with the experimentally measured analyte distribution (pixel values). At later time *t*_*n*_, the analyte spreads further along the channel.

For the infinite source systems, we expect that the concentration of the analyte (hence its color) at the source (*x* = 0) to remain unchanged. On the other hand, the finite source gets depleted with time and the analyte concentration decreases (color becomes less dark, *i.e.*, higher pixel value). These characteristics are, of course, expected from the simulated results due to the boundary conditions. We observe the trends in experimental measurements as well (especially in the initial time steps) – see Fig. 5.

**Fig. 6:**
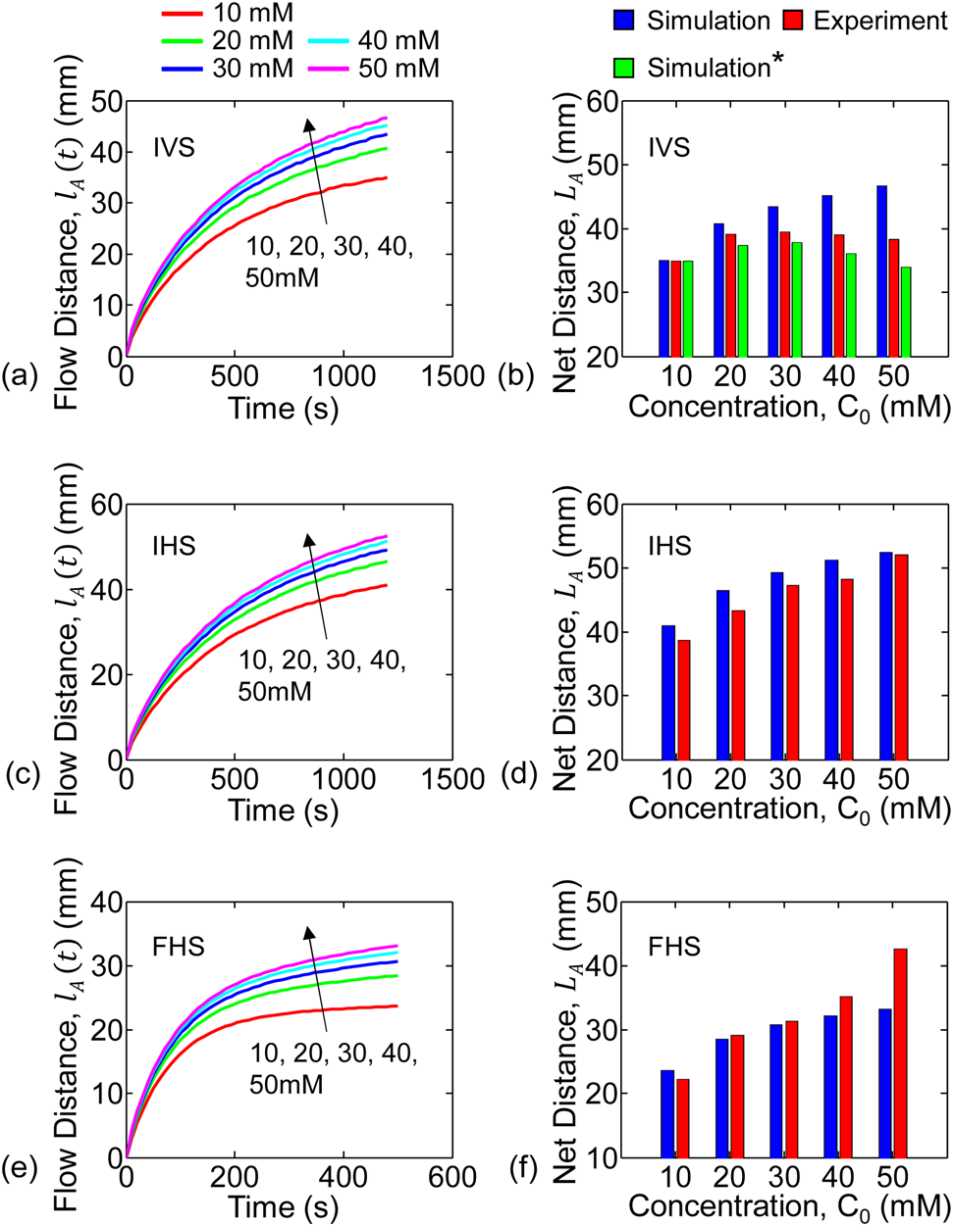
Numerically simulated analyte flow distances through the paper-channel are shown for various concentrations. Continuous analyte flow distance (*l*_A_(*t*)) *versus* time for IVS, IHS, and FHS are shown in (a, c and e). Also, the end-of-experiment net flow distance *versus* concentration for IVS, IHS, and FHS are summarized in (b, d and f). Here, the blue and red bar plots are from simulations and experiments, respectively. The green bar plots include gravitational effects in IVS system simulations.

The spatial profile of concentration observed at time *t*_1_ will further move along the channel over time (from *t*_1_ to *t*_*n*_). For the numerical simulations, we assume the analyte flow distance to be the position at which the concentration profile reaches the threshold *C*_th_ = 2 mM. The *C*_th_ has been chosen here to obtain a closer match in the net flow distance (*L*_A_) between simulations and experiments. The flow distance for each time step is combined to find the simulated analyte net flow-distance *versus* time. This is repeated for all the concentrations under study – see Fig. 6(a, c and e). Here, the results for *β* = 0 have been illustrated for comparative discussions. As observed in the simulation (Fig. 6(a and c)) and experimental (Fig. 3(a and c)) results, the flow-distance *versus* time characteristics for infinite sources (IVS, IHS) does not saturate as early as in the case of finite source (FHS). This is dominated by the time-series behaviour of water flow and velocity. Due to the finite volume, water velocity quickly decays towards zero and consequently slows down the analyte flow in case of FHS. Due to the same reason, the analyte in FHS flows slower and to a shorter distance than that in IVS or IHS. See water flow distance *l*_w_*vs.* time for the three configurations in the ESI document.

The total flow-distance found after 1200 s (IVS and IHS) or 500 s (FHS) for the different analyte concentrations are summarized in the bar plots in Fig. 6. The blue and red bar plots represents results from the numerical simulation and the experiments, respectively. The numerically predicted results are consistent with the experiments for IHS and FHS. Trends of both the time-series flow-distance, and the net flow for different concentration are consistently represented by the numerical results – this indicates that our hypothesis of dual transport-phase flow for this solution system is adequate. Overall, the characteristics observed determines the relationship between liquid sample concentration and flow distance. This relation can be used for other chemicals (with dual transport or two-phase flow) to identify the concentration.

In general, for a given setup (IVS, IHS or FHS), the water flow velocity (*v*_w_(*t*)) is expected to be consistent for repeated experiments. The *v*_w_(*t*) should be independent of the concentration used (due to the dilute solutions), as previously mentioned in Sec. 5.1. We do observe this consistency for IHS and FHS experiments (see Sec. S2 in the ESI† document). Therefore, using the fixed set of parameter values as listed in Table 2 give practically relatable numerical results for all concentrations for IHS and FHS. The net flow distance of analyte (*L*_A_) for IVS predicted by the numerical simulation (based on parameters in Table 2) does not follow the experiments – see blue and red bar plots in Fig. 6, respectively. This can be due to the combined effects of: (i) gravitational force on analyte, and (ii) the fact that the water flow velocity (*v*_w_(*t*)) for IVS varied among experiments. The effect of gravitational force can be modeled with non-zero *β*in eqn (4), as discussed earlier. We extracted the *v*_w_(*t*) profiles separately for each of the IVS experiments (Sec. S2†) and then did corresponding numerical analysis including graviational force on analyte – see Sections S2 and S3 in the ESI† document for full details. The resulting net flow distances for the various analyte concentrations are shown by the green bar plots (labeled as Simulation*) in Fig. 6(b). These results are more consistent with the experimental observations (red bar plots). It is unclear why there was observable inconsistencies among water velocities among the IVS experiments. Further study is needed to explain the effects of gravity in the IVS systems.

### 5.3. Flow distance *vs.* diffusion length

Conventionally, we do not expect concentration dependency of diffusion distance. It is therefore important to discuss the difference between the ‘diffusion distance’ and the ‘flow distance’ (*l*_A_). In the following, we will explain why the ‘flow distance’ shows concentration dependency, even though ‘diffusion distance’ does not.

**Fig. 7:**
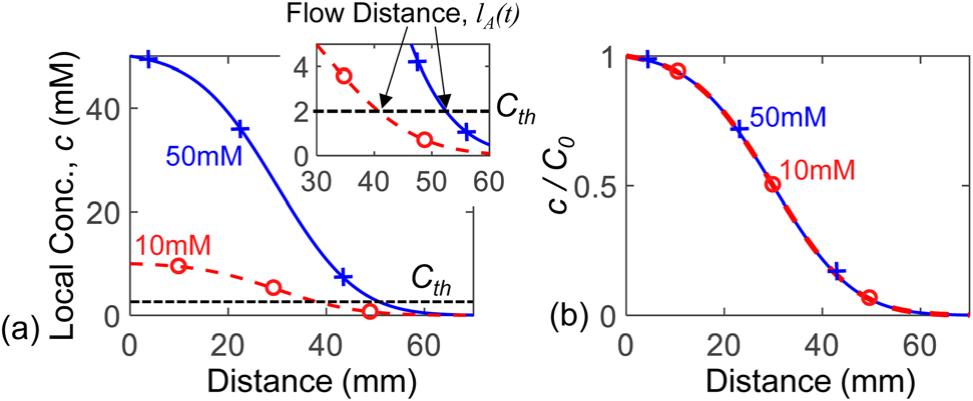
(a) Numerically simulated spatial profile of concentration through the paper-channel in IHS setup shown for 10 mM and 50 mM source concentration (*C*_0_) at *t* = 1200 s. The threshold *C*_th_ marks the flow distance *l*_A_ – see the zoomed-in plot in the inset. (b) The same plots are shown after normalization to respective source concentrations *C*_0_.

The flow distance at any time (*l*_A_(*t*)) is the distance as perceived by the intensity threshold in the experiment (*i.e.*, edge where the dark analyte front ends), or equivalently by a concentration threshold as explained in the numerical simulations results in Sec. 5.2. For example, in the IHS setup, the concentration profiles after *t* = 1200 s for 50 mM and 10 mM solutions are shown in Fig. 7(a). As per definition, the flow distance (*l*_A_) is the distance at which the local concentration goes below the threshold *C*_th_, which equivalently represents the intensity threshold. Clearly, the 50 mM solution travels more than the 10 mM source solution. Our results are, in fact, also consistent with the conventional ‘diffusion length’ concept. Within the diffusion length, *c*/*C*_0_ decreases by a fixed amount. For example, if we plot our results as ‘*c*/*C*_0_*vs.* distance’, as shown in Fig. 7(b), the curves for the 10 mM and 50 mM sources overlap. This indicates that the diffusion distance for different concentrations would be the same – however, the flow distance (*l*_A_) as seen by the observer would be concentration dependent.

## 6. Conclusion

In this work, we have experimentally and numerically analyzed the spatio-temporal wicking behavior of KMnO_4_ solutions through paper strips for different concentrations. In the solution chosen (KMnO_4_ in water), the analyte (*i.e.*, KMnO_4_) forms a weak bond with the solvent (water). As a result, its wicking through the paper shows two transport stages: a capillary-driven water flow, followed by a convection–diffusion mode analyte transport. We have conducted experiments and numerical modeling focusing on time-series progression of water and analyte flow through the paper-strip for different setup conditions (vertical and horizontal paper channel) and concentrations.

All the experiments were video recorded for time-series flow data extraction. We determined the water and analyte flow distance using our proposed video processing algorithm to ensure measurement consistency among experiments. We also explained the physics of the analyte flow through the paper-channels through experimental results and simulations. We have explained how the convection–diffusion equation with appropriate boundary conditions can model this diffusion-based analyte transport process. The water flow (concentration independent) contributes to a drift force on the analyte particles which accentuates the concentration dependent flow distance. We have explained through our model why the observed flow distance is concentration dependent, in contrary to concentration independent diffusion distance.

For horizontal paper channel setups (IHS and FHS), the average end-of-experiment final flow distance of the analyte (*L*_A_) showed monotonically larger values with increasing concentration. This is a key characteristic behavior that is specific to such two-transport phase flow (water assisted analyte flow) solution that can be useful for paper-based sensing devices. Concentration dependency would not be observed for fully dissolved solution with single transport phase flow which follows Lucas–Washburn like behavior. For the vertical setup (*i.e.*, IVS), on the other hand, the end-of-experiment final flow distance of the analyte (*L*_A_) increases for 10–30 mM concentrations, and then decreases for 40 mM and 50 mM solutions. We theorize that the gravitational forces on the solution in the vertical flow system causes this behavior. Indeed, our corresponding numerical results including gravitational effects consolidates this observation – this model, however, requires further development. Overall, the experimental and simulated data show good characteristic agreement. However, more studies are needed to improve the model to achieve a better quantitative match between simulations and experiments.

Paper-based detection has great potential for low-cost and point-of-care devices. Our work can be generalized to test and analyze other solute–solvent systems with two transport-stage flows. The numerical analysis can be further refined with more detailed 2D, transient models and by including environmental factors (*e.g.*, humidity, temperature, and evaporation). Deeper understanding of spatio-temporal behavior of analyte flow, such as that presented in this paper, will aid in developing and designing more complex and practically relevant paper-based sensors.

## Data availability

The extracted data (analyte flow distance with time) for all experiments are available at Zenodo at https://doi.org/10.5281/zenodo.14000186.

## Author contributions

Md. Saykat Hassan Sajib: data curation, validation, methodology, investigation, formal analysis, software, visualization, writing – original draft, writing – review & editing. Md. Sakif Rafid: data curation, validation, methodology, investigation, software, writing – review & editing. M. Ryyan Khan: conceptualization, project administration, funding acquisition, supervision, methodology, investigation, formal analysis, validation, software, writing – review & editing.

## Conflicts of interest

The authors declare that they have no known competing financial interests or personal relationships that could have appeared to influence the work reported in this paper.

## Supplementary Material

RA-015-D5RA02347E-s001

RA-015-D5RA02347E-s002

RA-015-D5RA02347E-s003

RA-015-D5RA02347E-s004
