# Supplementary material for: Experiment and modeling of concentration-dependent diffusion in a solution with dual transport-phase flow for paper-based sensing
Source: RSC Adv. 2025 Jun 4;15(23):18670–80. doi: 10.1039/d5ra02347e (PMC12134741; doi:10.1039/d5ra02347e)
Supplement: RA-015-D5RA02347E-s004 [file RA-015-D5RA02347E-s004.pdf]

Supplementary Information (SI) for RSC Advances.
This journal is © The Royal Society of Chemistry 2025

Supporting Document:

Experiment and modeling of concentration-dependent diffusion in a solution with dual transport-phase flow for paper-based sensing

Md. Saykat Hassan Sajib[a], Md Sakif Rafid[a], and M. Ryyan Khan[a]

[a] *Department of Electrical and Electronic Engineering, East West University, Bangladesh*
E-mail address: ryyan@ewubd.edu (M. Ryyan Khan)

S1. Experimental data

Remember that: IVS = Infinite-source with vertical strip; IHS = Infinite-source with horizontal strip; FHS = Finite-source with horizontal strip.

Each configuration-IVS, IHS, and FHS-was tested independently with repetitions across all concentrations to ensure consistency and reliability of the results (see Table S1).

Table-S1. Number of experimental repetitions conducted under varying concentrations (10-50mM) and experimental setups:

	10mM	20mM	30mM	40mM	50mM
IVS	9	9	9	9	9
IHS	4	4	4	4	4
FHS	4	4	4	4	4

A sample set of plots showing temporal progress of pixel value vs. distance is summarized in Fig. S1.

As listed in the Table-S1, multiple/repeated experiments were conducted for each concentration of each (IVS, IHS, or FHS) setup. The mean trend, as shown by the red lines in Fig. S2, was used for the final distance detection analysis. The measurements from each repetition are shown by the light color (cyan) lines in these figures.

The net analyte distance (L_A) versus concentration (C_0) plots shown in Fig. 3 of the main manuscript has been reproduced here in Fig. S3. The standard deviations have been added to the bar plot in Fig. S3. This indicates the variability among repeated experiments. Note that Fig. S2 gives a more detailed picture of this variation.

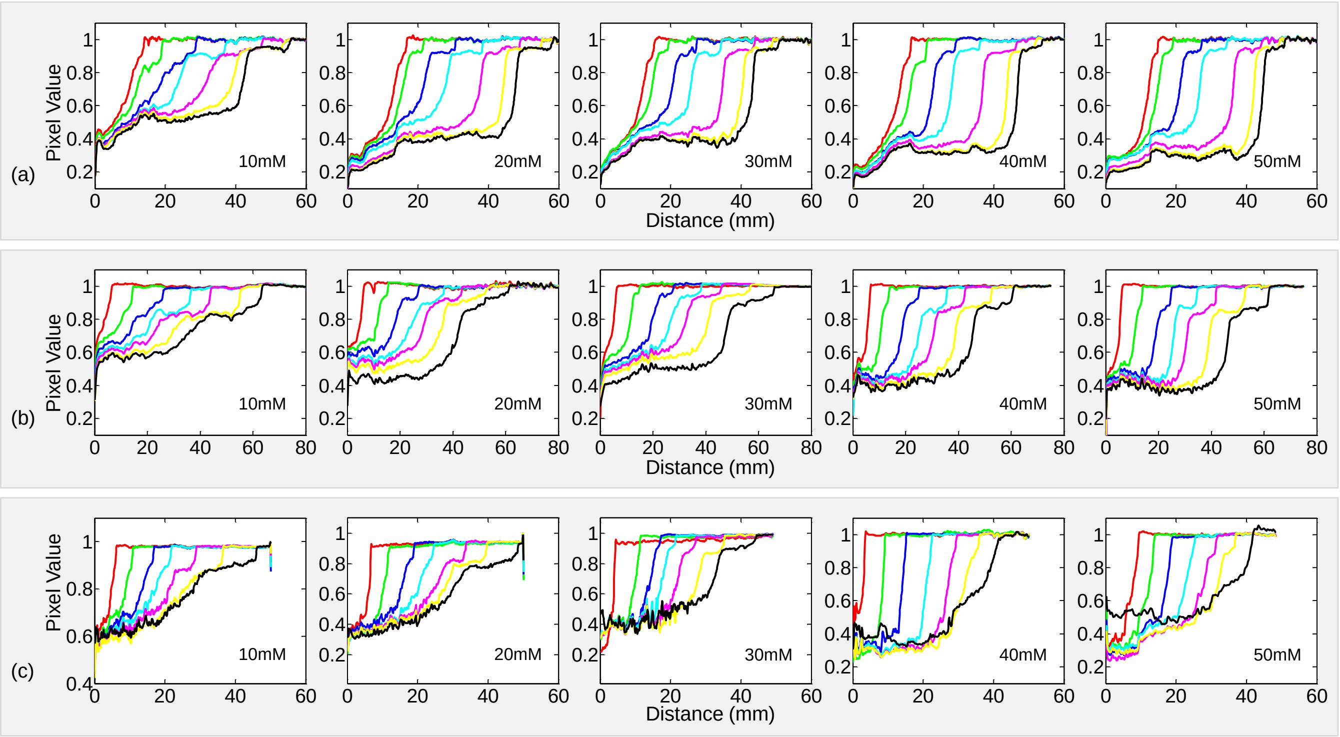

Fig. S1. A sample set of plots showing temporal progress of pixel value vs. distance is summarized here. These are used to find the analyte flow distance at different times for different concentrations for: (a) IVS (b) IHS (c) FHS.

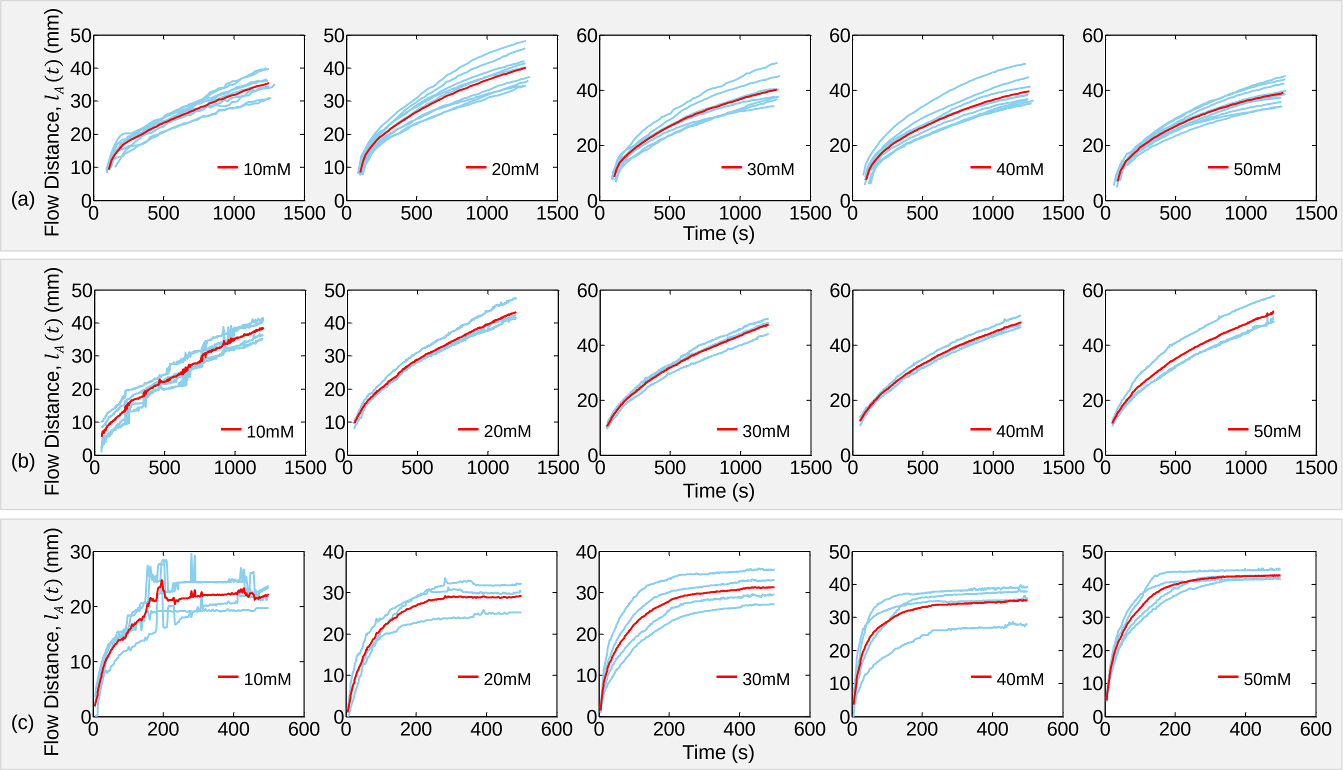

Fig. S2. The experimental results show the distance as a function of time for different concentrations: (a) IVS (b) IHS (c) FHS. The light blue lines represent separate runs of the experiment. The red lines represent the mean trend.

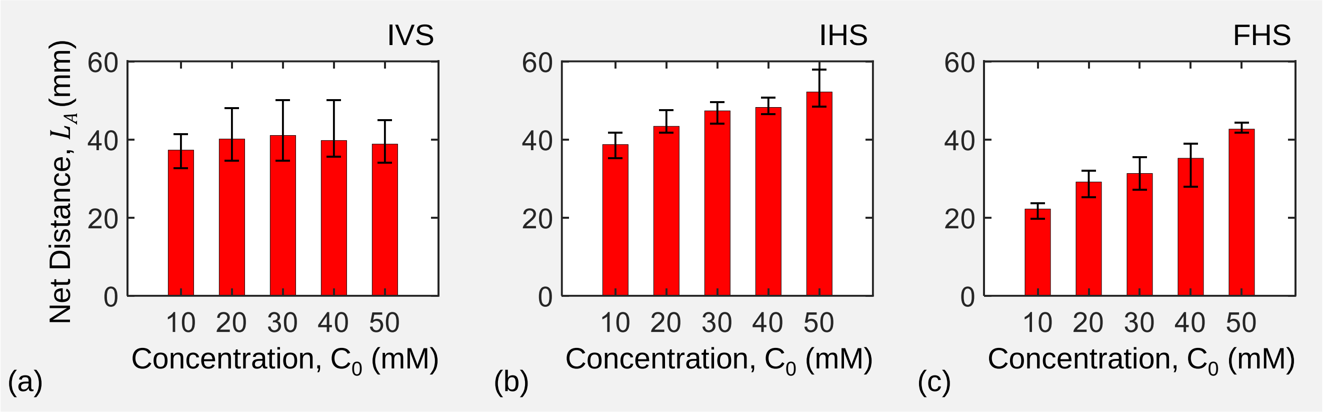

Fig. S3. The bar plots show the net distance as a function of concentrations: (a) IVS (b) IHS (c) FHS. Red bars represent the average net distance traveled, while error bars indicate the standard deviation from repeated measurements.

S2. Water velocity analysis

Instead of the threshold method used to detect the analyte flow front (as discussed in the main manuscript), we detect the transition in pixel value (f_{PV}) associated with the water flow front and follow it through the different time frames. We observe that there is a small, yet sharp step in f_{PV} vs position (x) curve where the water touches the dry paper strip. We smooth (moving average with window size ~5) the $f_{PV}(x)$ curve and then find the derivative (df_{PV}/dx). The last peak position indicates the water flow front – see Fig. S4. This peak is tracked at each time-frame to find the water flow position $l_w(t)$ vs time (shown later in Figs. S5-S7). This method could have been used for the analyte flow front detection as well – in fact it works for the higher

concentrations of $KMnO_4$. However, the derivative method does not work consistently for low analyte concentration (e.g., 10mM) and hence we use the threshold method (as explained in the main manuscript).

For IHS, water flows approximately the same distance at different concentrations. A similar consistency is observed among FHS experiments. However, IVS shows inconsistent wicking behavior. Fig. S5 (left plot) shows water flow position $l_w(t)$ vs time for various experiments (different concentrations) – see light blue curves. The red curve gives the average. This is then fitted using $l_w = L_{w\infty}(1 - \exp(t/\tau))$ to find $L_{w\infty}$ and τ (see sec. 5.1 in main manuscript). The corresponding velocity $v_w = dl_w/dt$ is shown in Fig. S5 (right plot). A similar set of plots and analysis for FHS is shown in Fig. S6.

As mentioned earlier, the water flow was inconsistent among the experiments in IVS. In Fig. S7, water flow analysis plots are shown separately for each of the analyte concentration cases for IVS experiments. Correspondingly, the mean $L_{w\infty}$ and τ values for the different analyte concentrations are listed in Table-S2.

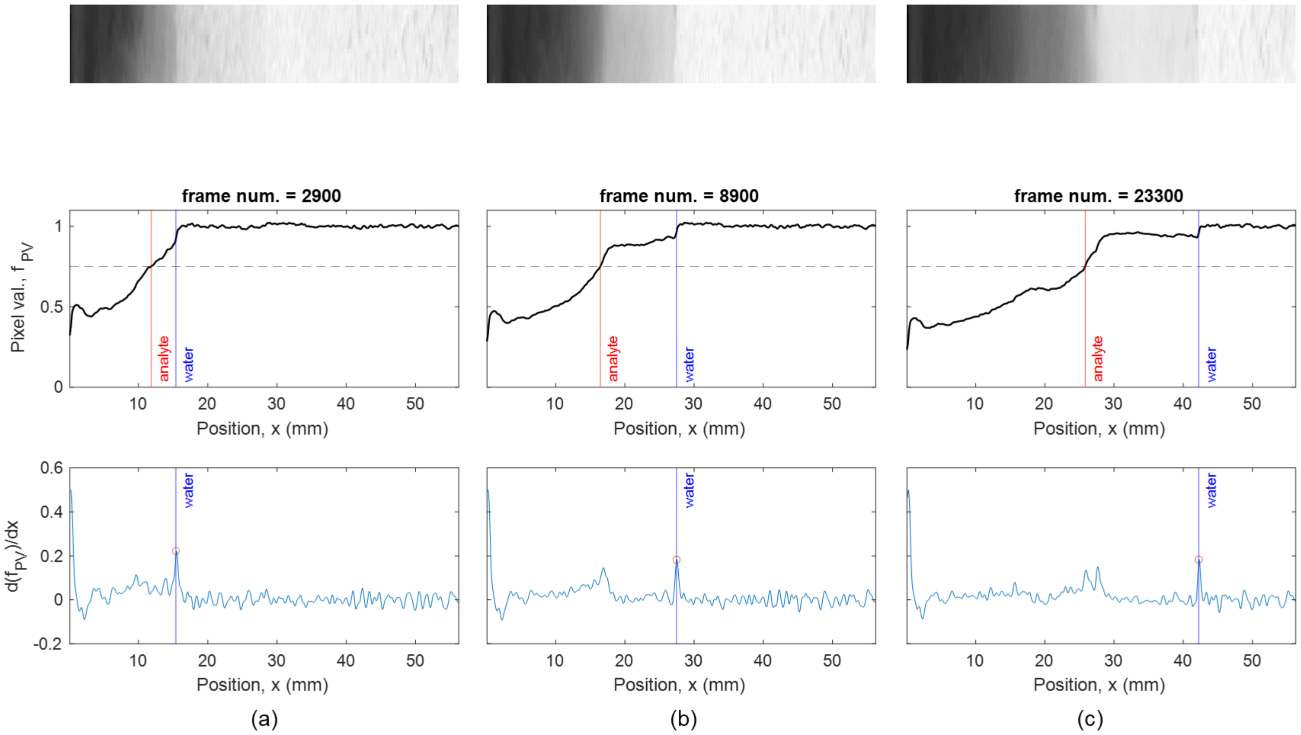

Fig. S4. Pixel value f_{PV} vs position x is shown for three different video frames (from one of the 10mM IVS experiments). The normalized derivative (df_{PV}/dx) is also shown. The last peak position indicates the water flow front.

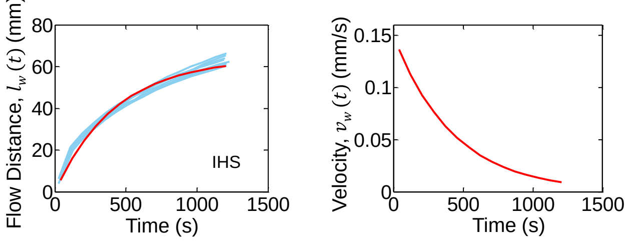

Fig. S5. Determining water velocity from experimental results by curve fitting for IHS experiment.

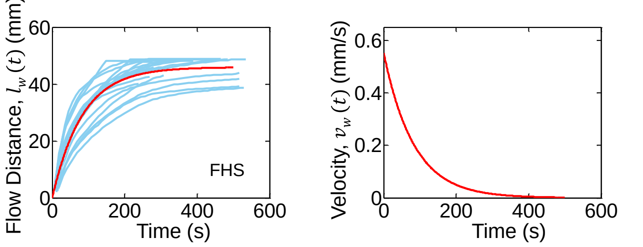

Fig. S6. Determining water velocity from experimental results by curve fitting for FHS experiment.

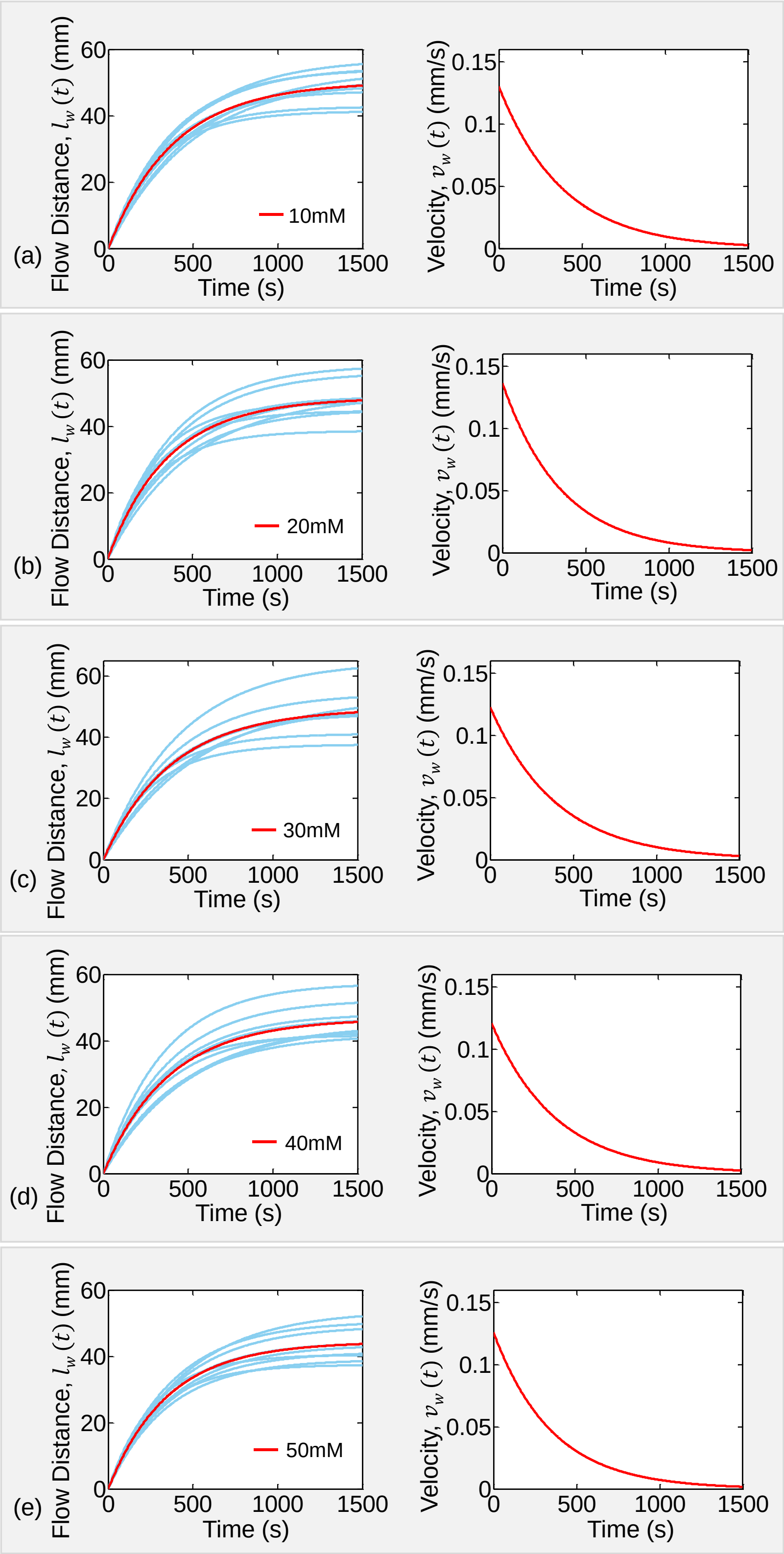

Fig. S7. Determining water velocity for different concentrations from experimental results by curve fitting for IVS experiment: (a) 10mM (b) 20mM (c) 30mM (d) 40mM (e) 50mM

Table-S2. Characteristic parameters of water flow fitted to $l_w = L_{w\infty}(1 - \exp(t/\tau))$ are given below for different analyte concentrations in IVS experiments.

Analyte conc. (mM)	$L_{w\infty}$ (mm)	τ (s)
10	49.95	383.53
20	48.39	354.99
30	49.17	400.28
40	46.57	385.37
50	44.27	350.70

S3. Analyte flow analysis considering gravity for vertical channel (IVS)

In the main manuscript, we followed the convection-diffusion equation to represent the system for analyte flow:

$$\frac{\partial c}{\partial t} = D\frac{\partial^2 c}{\partial x^2} - v(t)\frac{\partial c}{\partial x} - P(t) \tag{S1}$$

Here, the velocity of the analyte particle was related to the water velocity as $v(t) = \alpha v_w(t)$, and $P(t) = 0$. For IVS systems, as the paper-strip is vertically placed, gravity will affect both the water flow and analyte flow. Water flow and its velocity is found from experimental data – we do observe that water flow distance is shorter in case IVS systems, which can be associated to the effect of gravity on water flow. Equation (S1) represents the analyte flow, and it should be modified to include the gravitational force on the analyte particles. As per the IVS setup, gravity will work in the opposite direction of $v(t)$:

$$\frac{\partial c}{\partial t} = D\frac{\partial^2 c}{\partial x^2} - (\alpha v_w(t) - \beta C_0)\frac{\partial c}{\partial x} \tag{S2}$$

To the zeroth order, we assume the effect of the gravitational force to be proportional to the analyte source concentration C_0. Fig. 6(b) from the main paper is reproduced here for convenience. The green bar plots labeled as " Simulation* " have been found following equ. (S2). The parameter values are: $D = 0.07$ mm²/s, $\alpha = 0.53$, $\beta = 2.1 \times 10^{-4}$ mm/s/mM.

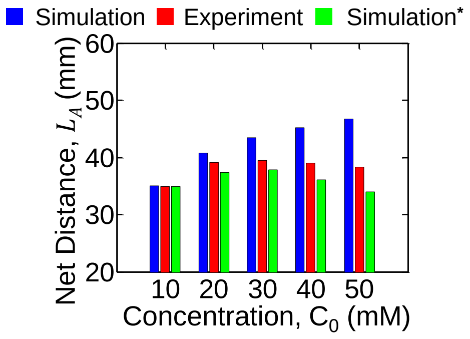

Fig. S7. Net analyte distance for various concentrations in IVS system.

S4. Mesh Analysis

We performed a mesh-time sensitivity analysis to evaluate the effects of time step (Δt) and spatial resolution (Δx) on the net flow distance for all three systems: IVS, IHS, and FHS (see Figs. S8–S10). The results showed that the time step had no significant effect on the net flow distance (for several orders of magnitude of Δt). However, the spatial resolution (Δx) did influence the results. We reduced Δx until the results (L_A) and error was stabilized and chose that Δx for our final simulations. Note that, the %-errors shown in these figures are with respect to the L_A -values found at the lowest (Δx, Δt) combination tested.

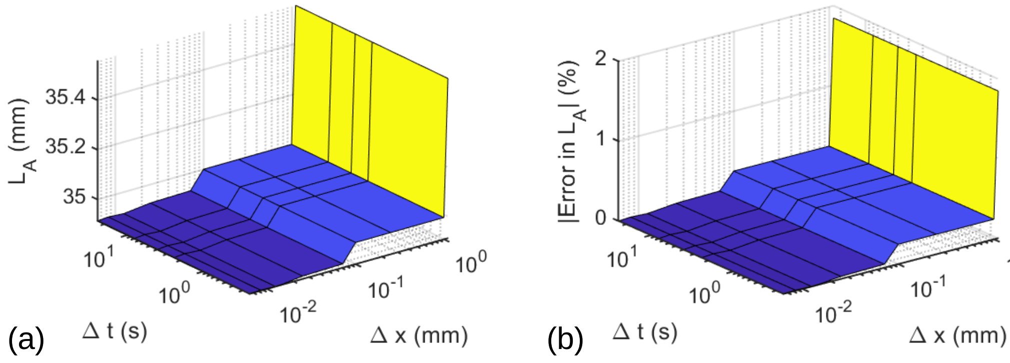

Fig. S8. Mesh and time sensitivity analysis for the IVS system. (a) Variation of the computed length with respect to spatial resolution and time step (b) Corresponding absolute percentage error in net distance relative to the reference solution.

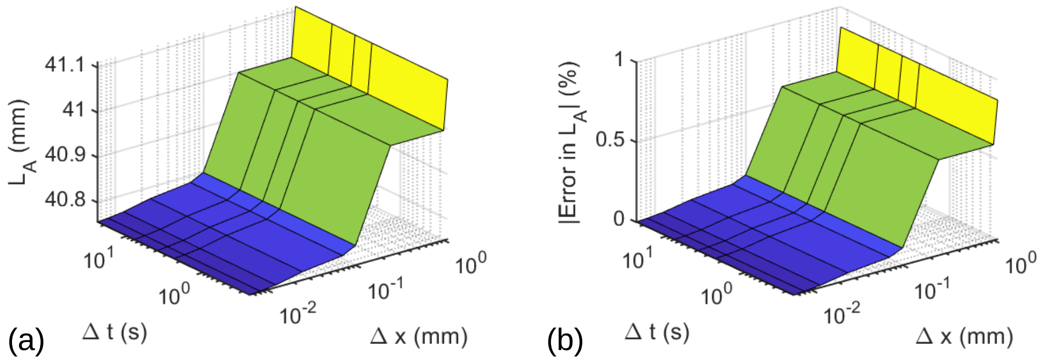

Fig. S9. Mesh and time sensitivity analysis for the IHS system. (a) Variation of the computed length with respect to spatial resolution and time step (b) Corresponding absolute percentage error in net distance relative to the reference solution.

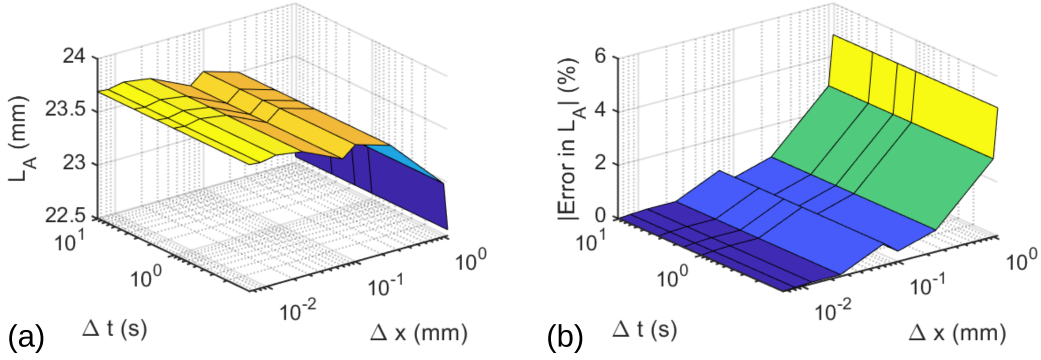

Fig. S10. Mesh and time sensitivity analysis for the FHS system. (a) Variation of the computed length with respect to spatial resolution and time step (b) Corresponding absolute percentage error in net distance relative to the reference solution.

S5. Video: Analyte and water flow distance extraction

Sample time-series analyte and water flow distance extraction videos are given as additional supplementary materials.

A step-by-step data extraction process is illustrated in Fig. S11(a) for a single time frame, demonstrating how the flow front positions of the analyte and water are identified through spatial calibration, image frame cropping, and grayscale image analysis. The final flow distances, determined from the last frame of the video, is in Fig. S11(b). This shows the complete progression of the analyte and water fronts over time.

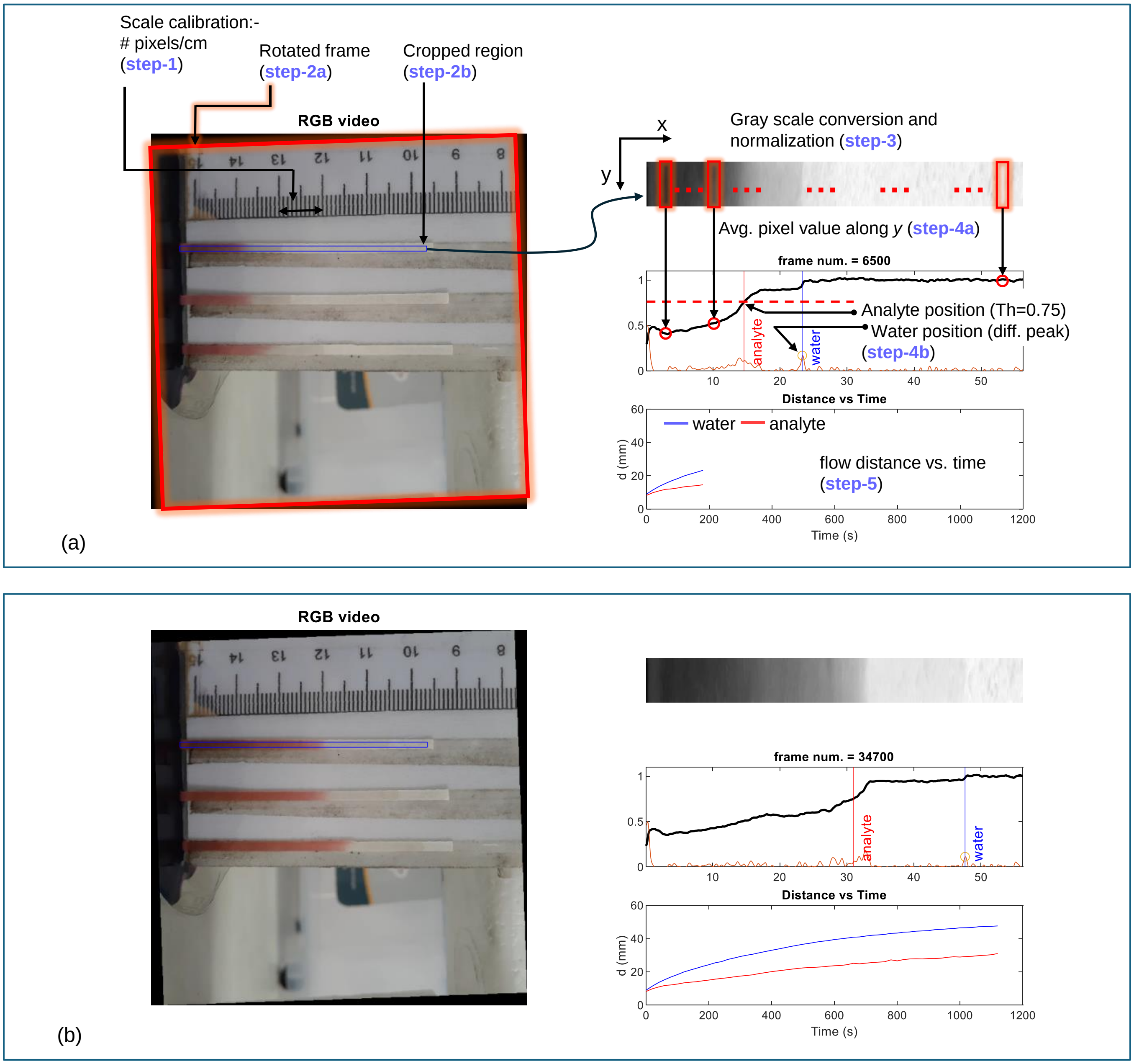

Fig. S11. Data extraction process. (a) Step-by-step image processing workflow for flow distance extraction from video frames (b) Final analysis result from the last video frame showing the detected positions of water and analyte fronts along with their respective time-resolved flow distance plots.
